# Proteasomal degradation of intracellularly expressed Amblyomin‐X limits suicide gene therapy potential in melanoma cells

**DOI:** 10.1002/2211-5463.70224

**Published:** 2026-04-02

**Authors:** Victor Dal Posolo Cinel, Carlos DeOcesano Pereira, Aline Ramos Maia Lobba, Melissa Regina Fessel, Ana Marisa Chudzinski‐Tavassi

**Affiliations:** ^1^ Centre of Excellence in New Target Discovery Instituto Butantan São Paulo Brazil; ^2^ Molecular Biology Universidade Federal de São Paulo Brazil; ^3^ Innovation and Development Laboratory Instituto Butantan São Paulo Brazil

**Keywords:** amblyomin‐X, cancer, gene therapy, Kunitz, melanoma, proteasome

## Abstract

Amblyomin‐X is a complex Kunitz‐type protease inhibitor from the tick *Amblyomma sculptum* , with selective pro‐apoptotic effects in tumor cells. While promising when applied exogenously, its large‐scale recombinant production is hindered by structural complexity. This study evaluated the feasibility of expressing Amblyomin‐X via a suicide gene therapy approach and investigated its intracellular fate following gene delivery in human melanoma and nontumoral cell lines. Amblyomin‐X transcripts were equally detected in both cell types; however, protein detection was restricted to nontumoral cells. Thus, we examined the mechanisms underlying Amblyomin‐X degradation in melanoma cells. Mutagenesis of predicted ubiquitination/SUMOylation sites failed to restore protein detection, whereas inhibition of proteasome activity with MG132 restored Amblyomin‐X detection. This confirmed the involvement of a proteasome‐dependent mechanism that limits protein stability and consequently its cytotoxic activity when intracellularly produced in melanoma cells. These findings indicate that proteasomal degradation represents a tumor‐specific barrier to the intracellular expression of Amblyomin‐X in human cancer cells. Addressing this limitation could enable the potent activity of Amblyomin‐X to be harnessed through gene‐based anticancer strategies. Furthermore, these findings advance the understanding of how structurally complex Kunitz‐type proteins with unstructured regions may behave in the context of gene therapy.

## Graphical abstract

This study explores the feasibility of expressing the antitumoral protein Amblyomin‐X through a suicide gene therapy approach and investigates its intracellular fate after gene delivery. Although the gene is efficiently expressed, melanoma cells rapidly degrade the Amblyomin‐X protein via proteasome activity. This tumor‐specific barrier highlights a key challenge for Amblyomin‐X–based gene therapy.

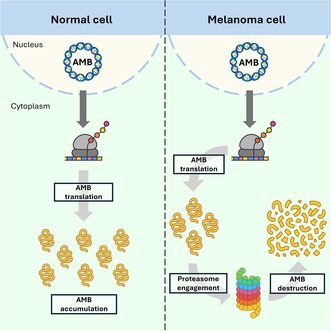

**Abbreviations**
AMB: amblyomin‐X
ATCC: American Type Culture Collection
BCA: bicinchoninic acid
COX8A: cytochrome c oxidase subunit 8A
D‐box: destruction box motif
DMEM: Dulbecco's modified Eagle medium
DMSO: dimethyl sulfoxide
eGFP: enhanced green fluorescent protein
FBS: fetal bovine serum
GAPDH: glyceraldehyde‐3‐phosphate dehydrogenase protein
HCS: high‐content screening
HEPES: 4‐(2‐hydroxyethyl)‐1‐piperazineethanesulfonic acid
HRP: horseradish peroxidase
IgG: immunoglobulin G
MTT: 3‐(4,5‐dimethylthiazol‐2‐yl)‐2,5‐diphenyl tetrazolium bromide
PBS: phosphate‐buffered saline
PenStrep: penicillin–streptomycin
PEST: proline–glutamic acid–serine–threonine–rich motif
RNase: ribonuclease
RT‐qPCR: reverse transcription quantitative polymerase chain reaction
SDS: sodium dodecyl sulfate
SUMO: small ubiquitin‐like modifier
TFPI: tissue factor pathway inhibitor

Cancer remains one of the most impactful diseases worldwide, responsible for approximately 9.7 million deaths in 2022 alone [1]. Despite advancements in conventional therapies, including surgery, chemotherapy, radiotherapy, immunotherapy, hormonal therapy, and photodynamic therapy, their effectiveness is frequently limited by issues such as nonspecific toxicity, resistance mechanisms, and tumor recurrence [2, 3]. By enabling the direct introduction of therapeutic or cytotoxic genes into cancer cells, gene therapy emerges as a targeted and potentially less harmful alternative to traditional cancer therapies [4]. Among anticancer gene therapy approaches, suicide gene therapy, based on introducing genes coding for cytotoxic proteins into tumor cells, has shown significant therapeutic potential [5, 6]. This strategy is particularly relevant for tumors that are poorly responsive to current treatment options, such as metastatic melanoma, where novel approaches are urgently needed [7, 8].

Amblyomin‐X (AMB) is a complex Kunitz‐type recombinant protein with antitumor properties, identified in the transcriptome of the salivary glands of the *Amblyomma sculptum* tick [9]. It shares structural similarity with the tissue factor pathway inhibitor (TFPI), a physiological inhibitor of factor Xa, and displays anticoagulant activity [10]. This molecule has demonstrated selective pro‐apoptotic activity in a variety of tumor cell lines, such as renal adenocarcinoma, pancreatic adenocarcinoma, pediatric anaplastic ependymoma, and melanoma, inducing tumor cell death through endoplasmic reticulum stress, proteasome inhibition, mitochondrial dysfunction, and caspase activation, while exhibiting minimal cytotoxicity in nontumor cells [11, 12, 13, 14]. Uptake studies also demonstrate preferential internalization by tumor cells via a cholesterol and PI3K‐dependent route [15, 16].

Preclinical studies have demonstrated that AMB promotes tumor mass reduction and is excreted renally with no observable systemic toxicity in animal models [17]. In a recent translational study, systemic administration of AMB led to significant tumor reduction in a spontaneous melanoma model in horses [18]. These findings reinforce the therapeutic relevance of AMB and support its continued development as an antitumor agent.

Despite its promising antitumor profile, large‐scale production of recombinant AMB poses technical challenges. These are mainly due to its structural complexity, including disulfide‐rich folding and an unstructured C‐terminal region, which complicate bacterial expression and reduce production yields. Moreover, the need for oxidative refolding steps further limits its scalability and the ability to obtain sufficient quantities for future applications. To address these challenges, a suicide gene therapy strategy based on plasmid‐driven expression of AMB under a strong constitutive promoter has been explored.

In this study, human SK‐MEL‐28 melanoma cells were used as the primary model to analyze AMB expression and cellular responses, and HEK‐293 T cells were included as a nontumor reference. Given the reported involvement of mitochondrial pathways in AMB's mechanism of action, AMB constructs with or without a mitochondrial targeting sequence were assessed. Within this framework, the present study was designed to evaluate the feasibility of expressing AMB via a suicide gene approach in melanoma cells and to investigate its intracellular fate following gene delivery.

## Methods

### Construction of plasmid vectors

Plasmids were based on the commercial mammalian expression vector pcDNA3.1 (#V79020; Thermo Fisher Scientific, USA) and were designed to evaluate the effects of AMB or eGFP expression with or without mitochondrial targeting, given the previously described role of AMB in mitochondrial dysfunction. Vector designs were generated using SnapGene 5 (Dotmatics, UK). The full‐length AMB and eGFP coding sequences were synthesized and inserted into pcDNA3.1 immediately downstream of the KpnI site by homologous recombination by the company GeneOne (Brazil). For mitochondrial targeting, the coding sequence of the signal peptide derived from the human cytochrome c oxidase subunit 8A (COX8A) precursor was inserted upstream of the AMB coding sequence by homologous recombination, generating the mitochondria‐targeted construct, while the nontargeted construct lacked specific targeting signals. An empty pcDNA3.1 backbone without a transgene was used as control (p_empty). The exact nucleotide sequences of the AMB coding region and mitochondrial targeting sequence are provided in Table 1. Construct identity was confirmed by Sanger sequencing provided by the manufacturer (GeneOne, Brazil).

**Table 1:** Nucleotide sequences of Amblyomin‐X and the mitochondrial targeting signal.

Amblyomin‐X	ATGGCCAACTCAAAGGCCGTGTGCAACCTGCCCAAGCTGGCCGGCGACGAGACCTGTAGCAACAAGACAGAAATCAGATGGTACTATAACGGCACAGCCTGCGAGGCCTTCATTTTCAAGGGCTGTGGGGGCAACGACAACAACTTTGACAGGGTGGACGATTGCCAGAGGCTGTGCGAGGAGCAGACCCACTTCCACTTCGAGTCTCCCAAGCTGATTTCGTTTAAGGTGCAGGACTACTGGATCCTGAACGATATCATGAAGAAAAACCTCACAGGTATTAGCCTGAAATCCGAGGAAGAGGACGCCGATTCCGGCGAAATTGACTGA
Mitochondrial targeting signal	ATGAGCGTGCTGACACCTCTGCTGCTGCGGGGACTGACAGGCAGTGCCAGGAGGCTGCCCGTGCCTCGGGCCAAGATCCACAGCCTGGGCGACCCCCCCGTGGCTACC

*Note:* All sequences are shown 5′ → 3′.

To enable monitoring of transfected cells, a pcDNA3.1 derivative co‐expressing the fluorescent protein E2‐Crimson was generated. The pcDNA3.1 backbone was modified (GeneOne, Brazil) to replace the neomycin/G418 resistance cassette with the E2‐Crimson coding sequence, yielding the pcDNA3.1‐E2‐Crimson recipient vector. E2‐Crimson is an auto‐fluorescent far‐red fluorescent protein derived from the DsRed‐Express2 variant, characterized by excitation and emission maxima at approximately 611 nm and 646 nm, respectively [19].

In order to generate plasmids co‐expressing AMB and E2‐Crimson, AMB coding sequences (nontargeted or mitochondrial‐targeted) were subsequently subcloned from the original AMB plasmids into pcDNA3.1‐E2‐Crimson using HindIII and NotI restriction enzymes (Thermo Fisher Scientific, USA), followed by T4 DNA ligase (Invitrogen, USA) mediated ligation. Ligated plasmids were transformed into competent bacteria, amplified, and purified by miniprep. Construct identity was verified by diagnostic restriction enzyme digestion (HindIII and XhoI) and agarose gel electrophoresis. As a control for the use of the E2‐Crimson plasmid, the vector expressing E2‐Crimson alone was used and referenced as ‘p_empty_E2’.

### Human cell culture

The cells used in this study were SK‐MEL‐28 (RRID: CVCL_0526, ATCC, USA), human melanocytes derived from a patient with malignant melanoma, and HEK‐293 T cells (RRID: CVCL_0063, ATCC, USA), nontumoral human epithelial‐like cells isolated from the kidney of a fetus. All cells were cultured in flasks in an incubator at 37 °C with 5% CO_2_, using Dulbecco's modified Eagle medium High Glucose (DMEM, Sigma‐Aldrich, USA) supplemented with 10% fetal bovine serum (FBS, Sigma‐Aldrich, USA) and 1% PenStrep antibiotics (Gibco, USA). All cell lines were routinely tested and confirmed to be mycoplasma‐free. For all experiments, on the day before treatment/transfection, 4000 SK‐MEL‐28 cells per well or 8000 HEK‐293 T cells per well were seeded in black 96‐well plates (#655986; Greiner, USA). For 6‐well plates (#CLS3516; Corning, USA), 200,000 SK‐MEL‐28 cells per well or 300,000 HEK‐293 T cells per well were seeded. For HEK‐293 T seeding, the plates were precoated with a thin layer of 0.1% Poly‐L‐Lysine (#P8920; Sigma‐Aldrich, USA) to enhance adhesion.

### Cell transfection

Lipofectamine LTX & Plus reagent (#15338100; Thermo Fisher, USA) was used for the transfection of both SK‐MEL‐28 and HEK‐293 T cells. In all transfection assays, reagents proportion was kept as 100 ng of plasmid, 0.1 μL of the Plus reagent and 0.5 μL of Lipofectamine LTX. The solutions containing the plasmid and reagents were prepared in Opti‐MEM medium (#31985070; Thermo Fisher, USA) and kept at room temperature for 30 min before transfection. The resulting solution was then added to the cells, and the plates were incubated at 37 °C with 5% CO_2_. A quantity of 100 ng of plasmid was used per well in 96‐well plates and 3 μg of plasmid per well in 6‐well plates. Transient plasmid transfection was selected to investigate how cells handle AMB upon gene delivery under conditions that minimize long‐term adaptation of the cell population.

### Verification of eGFP expression by fluorescence microscopy

To verify the expression of plasmids encoding eGFP, SK‐MEL‐28 and HEK‐293 T cells were transfected, and 3‐day post‐transfection, the cells were stained with Hoechst‐33342 solution (1 : 3000, #62249; Sigma‐Aldrich, USA) and visualized using the High‐Content Screening ImageXpress Micro‐Confocal System (HCS, Molecular Devices, USA). For cell counting, the following masks were applied in the MetaXpress software: The total cell count mask was configured to consider only Hoechst staining (λExc = 350 nm, λEm = 460 nm) located in the cell nuclei. The counting of eGFP^+^ cells was performed using a mask configured to detect eGFP staining (λExc = 502 nm, λEm = 530 nm) in any cellular portion. Transfection efficiency was calculated using the following formula: Transfection efficiency (%) = (eGFP^+^ cells/total cells) × 100.

### Total RNA and protein extraction

Total RNA and proteins were extracted from cell cultures using TRIzol reagent (#15596026; Thermo Fisher, USA). Briefly, after treatments, the culture medium was removed, cells were washed with PBS and then collected in cold TRIzol. Phase separation was performed using chloroform, and RNA and proteins were isolated according to the manufacturer's instructions. The RNA pellet was resuspended in RNase‐free water and further purified using the RNAspin Mini RNA Isolation Kit (#25–0500‐71; Ilustra, USA) according to the manufacturer's instructions. RNA concentration was measured by absorbance at 260 nm with a NanoDrop spectrophotometer (Thermo Fisher, USA). Proteins were resuspended in 2% SDS solution, and the quantification was performed using the Pierce BCA Protein Assay Kit (#23227; Thermo Fisher, USA).

### Reverse transcription of total RNA

Using total RNA isolated as described before, RNA was reverse transcribed into cDNA with SuperScript III First Strand Synthesis Mix kit (#18080051; Invitrogen, USA) according to the manufacturer's instructions on the Veriti 96‐well Fast Thermal Cycler (Applied Biosystems, USA). Oligo‐dT primers and random sequence primers were simultaneously used for the amplification.

### Verification of AMB expression by RT‐qPCR

AMB expression at RNA level was verified using the RT‐qPCR technique. For this, SK‐MEL‐28 and HEK‐293 T cells were transfected in 6‐well plates with either AMB‐expressing or empty plasmids. After 3 days of transfection, the cells were lysed, and total RNA was extracted, purified, and RNA was reverse transcribed into cDNA. Quantification by RT‐qPCR was performed using the Fast SYBR Green Master Mix reagent (#4385612; Applied Biosystems, USA), with the forward primer (5′ ATGGCCAACTCAAAGGCCGTG 3′) and reverse primer (5′ GTGGAAGTGGGTCTGCTCCTC 3′) targeting the AMB gene sequence.

### Protein expression verification by western blot

For western blot analysis, proteins were extracted from transfected cell cultures grown in 6‐well plates using the protein fraction recovered from TRIzol, as described in Section 2.5. Protein samples were resuspended in 2% SDS, mixed with sample buffer (2% SDS, 10% glycerol, 0.2 M 2‐mercaptoethanol and 0.01% bromophenol blue), and heated at 95 °C for 5 min before electrophoresis. Proteins were separated by SDS/PAGE and transferred to 0.45 μm nitrocellulose membranes.

Western blot was performed 3‐day post‐transfection to verify transgene expression. For primary antibody labeling, the membrane was incubated overnight at room temperature with the following antibodies: mouse anti‐GFP primary antibody (#sc‐9996; Santa Cruz, USA) at a 1 : 5000 dilution; rabbit anti‐GAPDH primary antibody (#sc‐25778; Santa Cruz, USA) at a 1 : 1000 dilution; mouse anti‐Ubiquitin primary antibody (#13–1600; Invitrogen, USA) at a 1 : 1000 dilution; rabbit anti‐AMB primary antibody (polyclonal, in‐house) at a 1 : 10,000 dilution. The secondary antibodies used were goat anti‐rabbit IgG HRP (#ab97051; Abcam, UK) and goat anti‐mouse IgG HRP (#ab97023; Abcam, UK), both at a 1 : 5000 dilution. Detection of the specific antibody‐bound proteins was performed using the Amersham ECL Start Western Blotting Detection Reagent (#RPN3243; Cytiva, USA).

### Mitochondrial activity assessment by MTT

Cell viability was assessed in SK‐MEL‐28 and HEK‐293 T cells by MTT assay (#475989; Sigma‐Aldrich, USA). After treating the cells, the culture medium was replaced with 0.5 mg·mL^−1^ MTT in culture medium. Following 3 hours incubation at 37 °C with 5% CO_2_, the MTT solution was removed, and dimethyl sulfoxide (DMSO, Merck, USA) was added to the wells to solubilize the formazan formed by the reduction of MTT by mitochondrial enzymes in viable cells. After solubilization, the plates were placed in a SpectraMax M5e device (Molecular Devices, USA) to measure absorbance at *λ* = 540 nm.

### Evaluation of apoptosis by caspase activity

Apoptosis induction in SK‐MEL‐28 cells was evaluated by assessing caspase 3 and 7 activity using HCS with the CellEvent Caspase‐3/7 Green reagent (#C10432; Thermo Fisher, USA). After cell treatment, the culture medium was replaced with a PBS buffer solution containing 5% v/v FBS, 3 μg·mL^−1^ Hoechst‐33342, and 2 μMm of the CellEvent Caspase 3/7 Green reagent. Cells were incubated for 30 minutes at 37 °C with 5% CO_2_. After incubation, the plates were imaged using the HCS, and the images were analyzed using MetaXpress software. To count the labeled cells, the following masks were applied in MetaXpress: the total cell count mask was configured to identify Hoechst staining (λExc = 350 nm, λEm = 460 nm) located in the nuclei. Similarly, the mask for counting cells with activated caspases 3 and 7 was set to identify fluorescent signals (λExc = 502 nm, λEm = 530 nm) exclusively present in the nuclear region (Hoechst‐33342 labeling).

### Cell death assessment by flow cytometry

To evaluate the fate of SK‐MEL‐28 cells at shorter time points, these cells were transfected with plasmids co‐expressing AMB and E2‐Crimson. The proportion of E2‐Crimson^+^ cells was assessed over time (1‐, 3‐, and 7‐day post‐transfection) using flow cytometry. Sample acquisition was performed using a FACSCanto II flow cytometer (BD Biosciences, USA), and data analysis was carried out with FlowJo 10.9 software (BD Biosciences, USA).

### *In silico* predictive analysis of destruction motifs

To investigate the presence of destruction motifs in the AMB protein sequence, several predictive analyses were performed using the following tools: GPS ARM (BioCuckoo, China) for identifying D‐box and KEN‐box destruction motifs; PESTfind (EMBOSS, Bioinformatics.nl, Netherlands) to detect PEST motifs; GPS UBER (BioCuckoo, China) for predicting ubiquitination sites; and GPS‐SUMO 2.0 (BioCuckoo, China) for predicting potential SUMOylation sites.

### Generation of nontargeted AMB plasmids with site‐directed mutations in lysine residues

To investigate the role of lysine residues in AMB stability, site‐directed mutations were introduced into the AMB sequence using the Phusion Site‐Directed Mutagenesis Kit (#F541; Thermo Fisher Scientific, USA). The PCR‐based mutation reaction was carried out using phosphorylated primers and the nontargeted AMB plasmid as a template. The primers (Table 2) were designed to introduce mutations at selected lysine residues predicted to be ubiquitinated, replacing them with arginine (K → R). Mutations were introduced at residues K5, K12, K22, K76, and K97. To confirm the success of the introduced mutations, Sanger sequencing was performed. Sequencing results were analyzed using the software geneious 9.0 (Dotmatics, UK).

**Table 2:** Primer sequences used for site‐directed mutagenesis.

Mutation	Primer forward	Primer reverse
K5R	TGGCCAACTCAAGGGCCGTGTGCAA	TGGTACCAAGCTTAAGTTTAAACGCTAG
K12R	GCAACCTGCCCAGGCTGGCCGGCGA	ACACGGCCTTTGAGTTGGCCATGGTACC
K22R	ACCTGTAGCAACAGGACAGAAATCAGA	CTCGTCGCCGGCCAGCTTGGGCA
K76R	CTGATTTCGTTTAGGGTGCAGGACTAC	CTTGGGAGACTCGAAGTGGAAGTG
K97R	GGTATTAGCCTGAGATCCGAGGAAGAG	TGTGAGGTTTTTCTTCATGATATCGTTC

*Note:* All sequences are shown 5′ → 3′. Mutations introduce K → R substitutions.

### Partial proteasome inhibition

Partial proteasome inhibition in SK‐MEL‐28 cells was performed using 5 μm of the inhibitor MG132 (#133407826; Calbiochem, USA) for 72 hours. The inhibitor concentration and exposure time were chosen to achieve partial proteasome inhibition while preserving sufficient cell viability and to allow adequate accumulation of the transgenic protein for detection within the same 72‐h post‐transfection window used for the other immunoblot assays.

To assess proteasome activity under these conditions, the fluorescent substrate suc‐LLVY‐AMC (#539142; Sigma‐Aldrich, USA) was used. After treatment, cells were washed twice with PBS and lysed in 100 μL of buffer containing 50 mm HEPES pH 7.4, 150 mm NaCl, and 1% Triton X‐100. Lysates were incubated at 37 °C for 30 min, followed by the addition of suc‐LLVY‐AMC to a final concentration of 100 μm. Fluorescence was measured using a SpectraMax M5e plate reader (Molecular Devices, USA) at λExc = 360 nm and λEm = 460 nm.

### Assessment of AMB production following proteasome inhibition

To investigate whether intracellular AMB was being degraded by the proteasome, SK‐MEL‐28 cells were transfected with AMB‐expressing plasmids and treated the following day with 5 μm MG132. After 72 h of treatment, cells were lysed, and protein extracts were collected. The samples were analyzed by SDS‐polyacrylamide gel electrophoresis and western blot to detect AMB expression.

### Statistical analysis

Experiments were performed in triplicate (*n* = 3 independent experiments), and results are expressed as mean ± SD unless otherwise stated. Comparisons between two groups used a two‐tailed unpaired *t*‐test. Comparisons among more than two groups used one‐way ANOVA with Tukey's *post hoc* test or with Dunnett's *post hoc* test against the negative control (untreated cells or empty plasmid). Analyses were performed in graphpad prism 9.0 (San Diego, USA), with *P* < 0.05 considered significant.

### Ethics statement

This study did not require ethics committee approval by the current regulations of the Universidade Federal de São Paulo (UNIFESP). Only commercially available human immortalized cell lines were used.

## Results

### Transfection efficiency

**Fig. 1:**
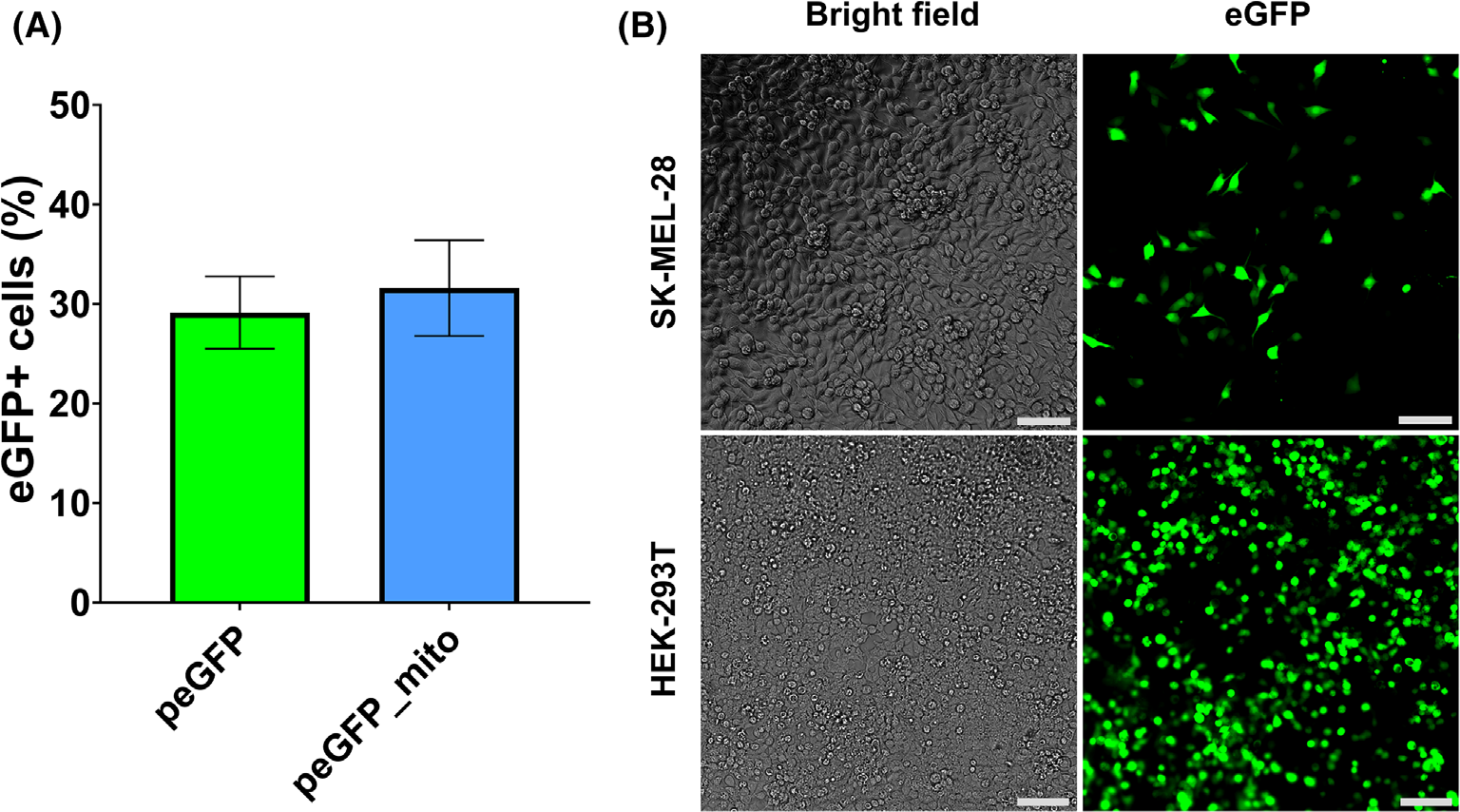
Transfection efficiency of SK‐MEL‐28 and HEK‐293 T cells with plasmids expressing eGFP. (A) Percentage of eGFP‐positive SK‐MEL‐28 cells transfected with plasmids encoding eGFP targeted to the mitochondria (peGFP_mito) or not (peGFP), evaluated by HCS 3 days after transfection. Data are mean ± SD of *n* = 3 independent experiments. (B) Representative microscopy images of SK‐MEL‐28 and HEK‐293 T cells transfected with the peGFP plasmid, illustrating the difference in overall transfection efficiency between the two cell lines; scale bars = 100 μm.

The transfection efficiency of nontargeted and mitochondrial‐targeted eGFP plasmids was evaluated in SK‐MEL‐28 and HEK‐293 T cells. In SK‐MEL‐28 cells, transfection rates were 29.15 ± 3.6% for the peGFP plasmid and 31.62 ± 4.81% for the peGFP_mito plasmid, showing no significant difference between them (Fig. 1A). In HEK‐293 T cells, both plasmids achieved transfection efficiencies above 95% (Fig. S1). Representative images of SK‐MEL‐28 and HEK‐293 T cells transfected with the peGFP plasmid (Fig. 1B) illustrate the marked difference in overall transfection efficiency between the two cell lines. Given the differences in transfection efficiencies across cell lines, interpretations are primarily made within each cell line.

### Verification of transgene expression

**Fig. 2:**
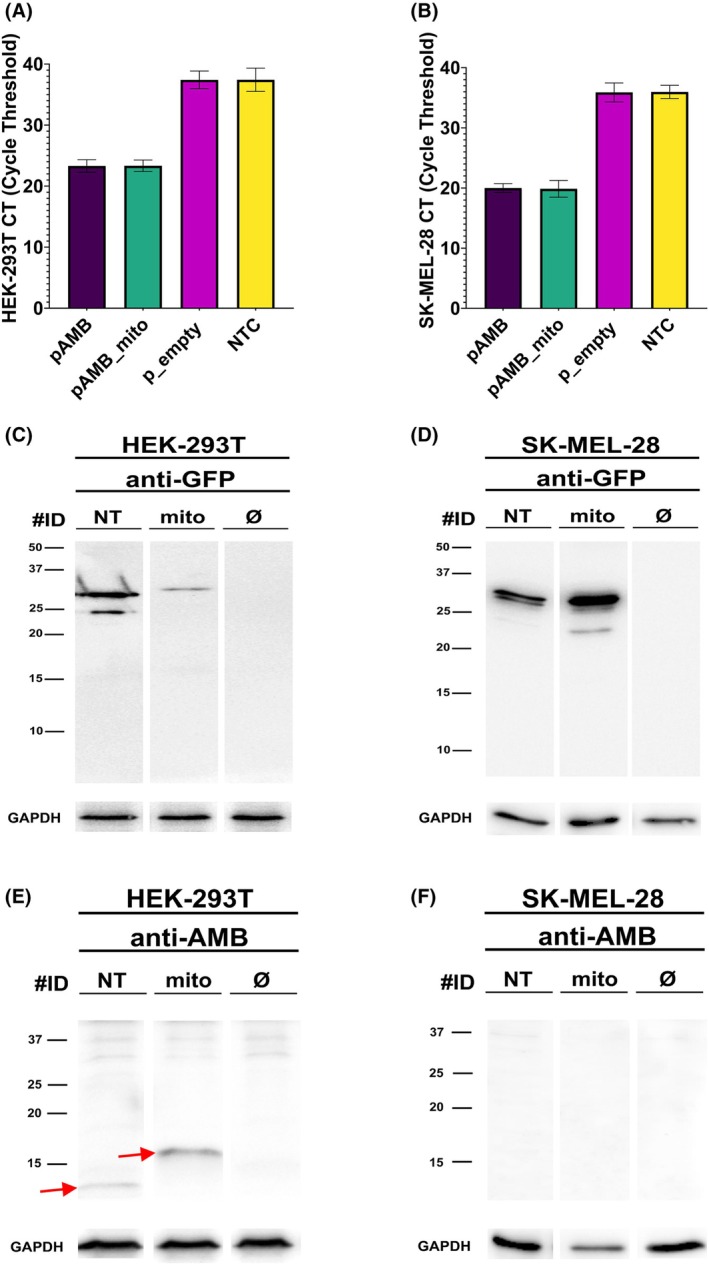
Verification of transgene expression at mRNA and protein levels 3‐day post‐transfection. (A, B) AMB mRNA levels determined by RT‐qPCR in HEK‐293 T (A) and SK‐MEL‐28 (B) cells transfected with the indicated plasmids in 6‐well plates. RT‐qPCR data are mean ± SD of n = 3 independent experiments. (C, D) Western blot detection of eGFP in protein extracts from HEK‐293 T (C) and SK‐MEL‐28 (D) cells transfected with eGFP‐expressing plasmids or empty vector in 6‐well plates. (E, F) Western blot detection of AMB in protein extracts from HEK‐293 T (E) and SK‐MEL‐28 (F) cells transfected with AMB‐expressing plasmids or empty vector in 6‐well plates. NT = nontargeted plasmid; mito = mitochondria‐targeted plasmid; Ø = p_empty plasmid. Arrows indicate the AMB bands at the expected molecular weights (~ 12.4 kDa for the NT form and ~ 16.2 kDa for the mito form). Nonadjacent lanes from the same gel are shown; all samples were run on the same blot. Molecular weight markers (kDa) are indicated on the left.

AMB at both transcript and protein levels were evaluated in SK‐MEL‐28 and HEK‐293 T cells following plasmid transfection. RT‐qPCR analysis confirmed comparable AMB RNA levels among all plasmid variants within each cell line (Fig. 2A,B). Western blot analysis revealed that eGFP protein was detected in both cell lines transfected with eGFP‐expressing plasmids, and, as expected, not in the p_empty controls (Fig. 2C,D). In contrast, AMB protein was detected only in HEK‐293 T cells transfected with AMB‐expressing plasmids (Fig. 2E), while no AMB signal was observed in SK‐MEL‐28 cells (Fig. 2F). The detected AMB bands correspond to ~ 12.4 kDa and ~ 16.2 kDa, the latter appearing at a higher molecular weight due to the presence of the mitochondrial targeting sequence.

Although the results demonstrate successful transcription of AMB in both cell lines, they highlight an important discrepancy in AMB protein detection between the nontumoral HEK‐293 T cells and the SK‐MEL‐28 melanoma cells (Fig. 2).

### Cell viability assessment by MTT

**Fig. 3:**
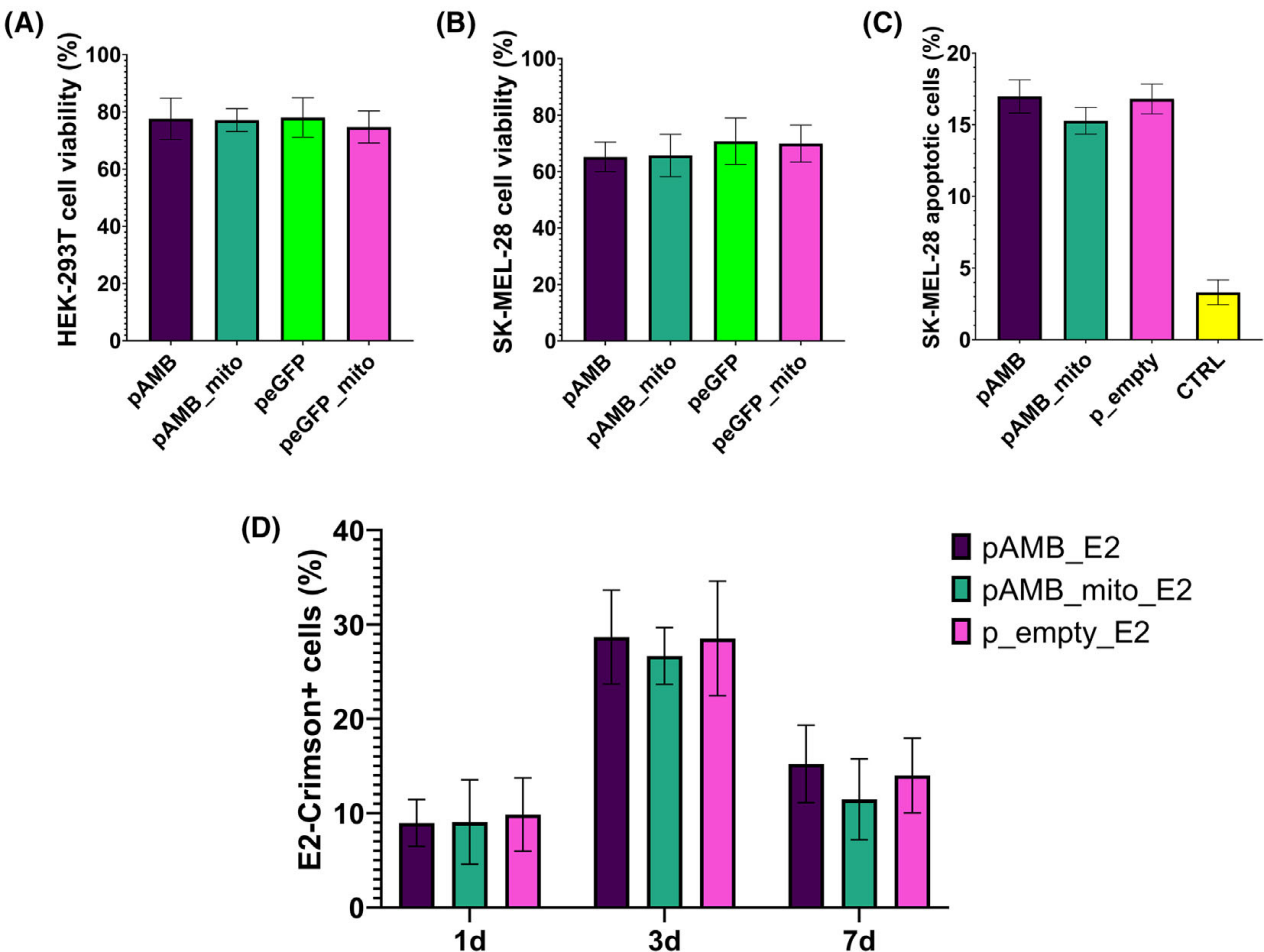
Evaluation of cell viability, apoptosis, and cell death at different time points post‐transfection. (A) Cell viability of HEK‐293 T cells was assessed via MTT assay 3‐day post‐transfection with AMB and eGFP‐expressing plasmids. (B) Cell viability of SK‐MEL‐28 cells was assessed via MTT assay 3‐day post‐transfection with AMB and eGFP‐expressing plasmids. MTT results are reported as normalized to nontransfected cells (set to 100%). (C) Proportion of apoptotic SK‐MEL‐28 cells assessed using the CellEvent Caspase 3/7 Green assay 3‐day post‐transfection. HCS results are reported as the percentage of total analyzed cells that were Caspase 3/7^+^; in panel C, CTRL refers to nontransfected cells. (D) Percentage of E2‐Crimson^+^ SK‐MEL‐28 cells measured by flow cytometry at 1‐, 3‐, and 7‐day post‐transfection with E2‐Crimson–AMB co‐expressing plasmids or the p_empty_E2 plasmid. Data are mean ± SD of *n* = 3 independent experiments.

The MTT assay indicated that transfection with eGFP‐expressing plasmids slightly reduced cell viability in both HEK‐293 T (Fig. 3A) and SK‐MEL‐28 cells (Fig. 3B), suggesting that the mild reduction may be related to the overexpression of a heterologous protein. A similar effect was observed in cells transfected with AMB‐expressing plasmids, indicating that AMB expression did not further impact cell viability under the tested conditions (Fig. 3).

### Measurement of caspase‐3/7 activity by HCS

In the apoptosis assay performed on SK‐MEL‐28 cells (Fig. 3C), a detrimental effect from the transfection process was observed, as indicated by the p_empty group, consistent with the results seen in the MTT assay with the eGFP plasmids. None of the plasmids encoding AMB resulted in a significant increase in apoptotic cells compared to the control, indicating that their transfection did not promote apoptosis under these conditions.

### Assessment of cell death via flow cytometry

The E2‐Crimson plasmids co‐expressing AMBs were utilized to evaluate the proportion of E2‐Crimson^+^ cells at 1‐, 3‐, and 7‐day post‐transfection in SK‐MEL‐28 cells. Since both genes are present on the same plasmid, all E2‐Crimson^+^ cells are expected also to express AMB. Thus, an indirect assessment of cell death was performed by comparing the proportion of E2‐Crimson^+^ cells between groups expressing AMB and the control plasmid p_empty_E2.

As shown in Fig. 3D, the proportion of E2‐Crimson^+^ cells remained consistent across all time points between the AMB‐expressing plasmids and the p_empty_E2. These findings suggest that AMB expression was not cytotoxic at any time point evaluated. Notably, the fraction of E2‐Crimson^+^ SK‐MEL‐28 cells reached a maximum of approximately 30% at 3‐day post‐transfection, indicating that this time point corresponds to the peak proportion of transgene‐expressing cells within the time points evaluated.

### *In silico* predictive analysis of destruction motifs

To assess whether nontargeted AMB and mitochondrial‐targeted AMB might be degraded after their production, the AMB sequence was analyzed using various degron prediction software tools.

No destruction motifs such as D‐box, KEN‐box, or PEST sequences were identified in the AMB sequence. However, the GPS‐UBER and GPS‐SUMO software predicted several lysine residues as potential sites for ubiquitination or SUMOylation (as shown in Table 3), which could lead to the protein's degradation via the proteasomal system.

**Table 3:** Predicted ubiquitination and SUMOylation sites in the AMB sequence.

Position	Peptide	Result
K5	MANSKAVCN	Ubiquitination
K12	CNLPKLAGD	Ubiquitination and SUMOylation
K22	TCSNKTEIR	Ubiquitination
K76	LICFKVQDY	Ubiquitination
K97	GISLKSEEE	Ubiquitination and SUMOylation

*Note:* The AMB sequence was analyzed using GPS‐UBER and GPS‐SUMO software for the prediction of lysine residues susceptible to post‐translational modifications. The table shows the position of lysine residues, the corresponding peptide sequence, and the predicted result (ubiquitination and/or SUMOylation).

### Construction of pAMB plasmids with site‐directed mutations in lysines

Based on the predictive analysis of ubiquitination and SUMOylation (Table 3), site‐directed mutations were introduced into the codons encoding lysines identified as potentially ubiquitinated or SUMOylated in the pAMB (nontargeted AMB plasmid) sequence, replacing them with arginines. These mutations were performed to determine whether it would be possible to prevent AMB degradation if the mechanism was mediated by ubiquitination or SUMOylation.

**Fig. 4:**
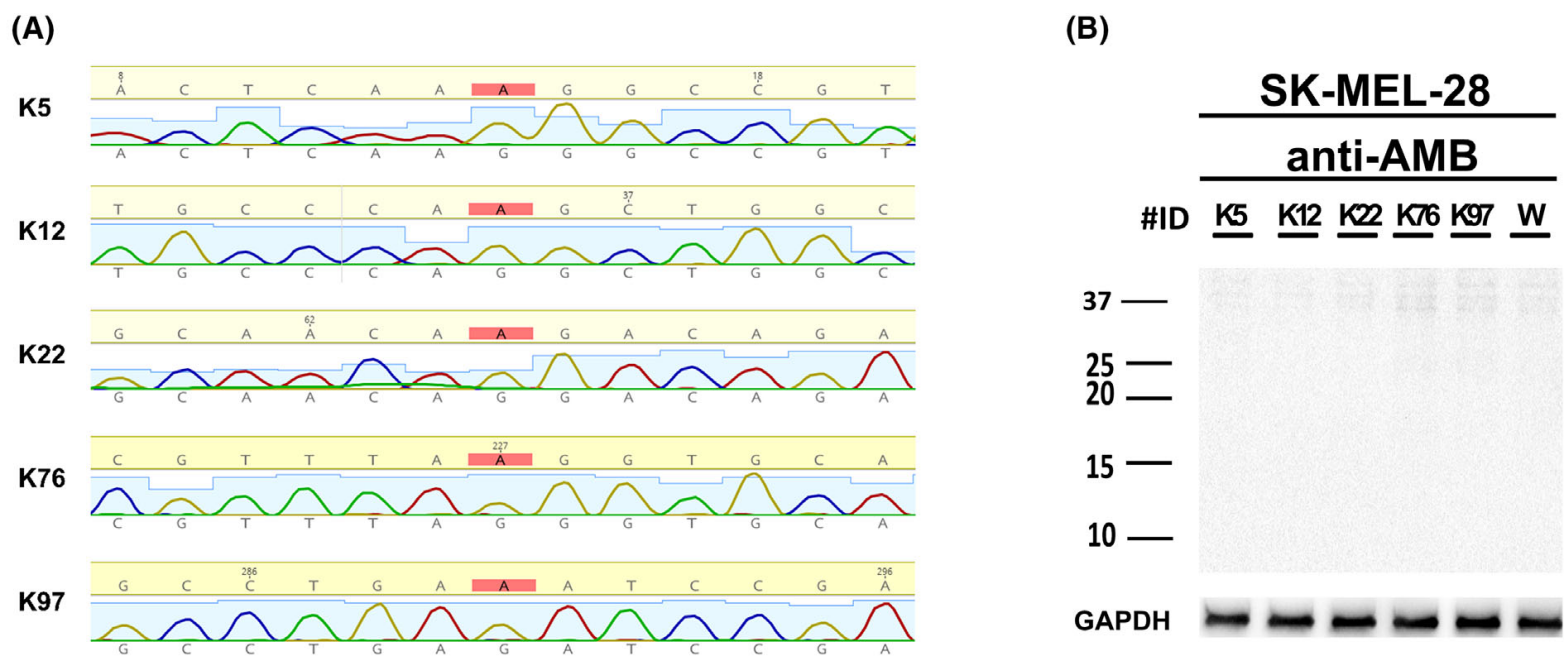
Analysis of lysine mutations and their effect on AMB protein stability in SK‐MEL‐28 cells. (A) Sanger sequencing analysis of the mutated pAMB plasmids. Constructs labeled K5, K12, K22, K76, and K97 correspond to single lysine‐to‐arginine substitutions at the indicated positions (i.e., K5R, K12R, K22R, K76R, and K97R) in the full‐length AMB sequence. (B) Western blot of SK‐MEL‐28 cells transfected with the non‐mutated AMB construct (W) and the mutants K5, K12, K22, K76, and K97, detected with the anti‐AMB antibody. Molecular weight markers (kDa) are indicated on the left.

Sanger sequencing confirmed the presence of the intended mutations (Fig. 4A), generating the following constructs: pAMB_K5, pAMB_K12, pAMB_K22, pAMB_K76, and pAMB_K97, in which the number refers to the position of the mutated lysine residue in the AMB protein.

### Verification of mutated AMB expression by western blot

To evaluate whether lysine mutations could prevent the nontargeted AMB protein from degradation in the intracellular environment, extracts from cells transfected with the mutated plasmids were analyzed by western blot.

Figure 4B demonstrates that lysine mutations were insufficient to stabilize the AMB protein in the cytoplasm, as no mutated AMBs were detected by western blotting of the respective cell extracts.

### Proteasome inhibition restores AMB detection in SK‐MEL‐28 cells

**Fig. 5:**
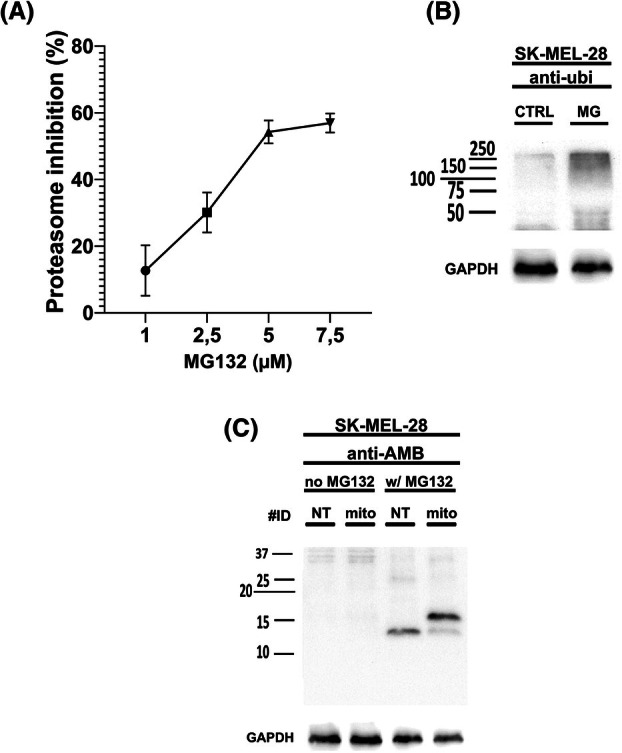
Evaluation of AMB protein levels in proteasome‐inhibited SK‐MEL‐28 cells. (A) Proteasome activity in SK‐MEL‐28 cells after treatment with increasing concentrations of MG132 for 72 h. Data are mean ± SD of *n* = 3 independent experiments. (B) Western blot detection of ubiquitin in protein extracts from SK‐MEL‐28 cells treated with 5 μm MG132. (C) Western blot detection of AMB in protein extracts from SK‐MEL‐28 cells transfected with nontargeted AMB (NT) or mitochondria‐targeted AMB (mito) plasmids, with or without treatment with 5 μm MG132. Molecular weight markers (kDa) are indicated on the left.

To further investigate whether AMB degradation occurred via the proteasomal pathway, a partial proteasome inhibition protocol was established in SK‐MEL‐28 cells using the MG132 proteasome inhibitor. A concentration of 5 μm MG132 for 72 hours reduced proteasome activity by approximately 58% (Fig. 5A) while maintaining ~ 50% cell viability, enabling the collection of cellular extracts. The effectiveness of proteasome inhibition was further confirmed by the accumulation of ubiquitinated proteins in MG132‐treated cells (Fig. 5B).

Proteasome inhibition also restored AMB detection in both the nontargeted and mitochondrial‐targeted constructs (Fig. 5C). The pAMB construct yielded a band corresponding to the expected molecular weight of ~ 12.4 kDa. In pAMB_mito‐transfected cells, two bands were observed: one at ~ 16.2 kDa, corresponding to AMB plus the mitochondrial signal peptide, and another at ~ 12.4 kDa, consistent with the cleaved mature form.

## Discussion

The use of AMB as a therapeutic molecule presents great potential for the treatment of solid tumors, as evidenced by the scientific literature [16]. In the case of melanoma, AMB has been shown to induce selective apoptosis in tumor cells without affecting nontumoral cells such as human fibroblasts [11]. However, despite its antitumor potential, large‐scale recombinant production of AMB remains challenging due to its structural complexity.

The gene therapy strategy employed in this study was based on the premise that intracellular production of AMB could overcome the limitations associated with recombinant AMB manufacturing and potentially reproduce the selective antitumor activity previously observed for the exogenous protein. This rationale also motivated the inclusion of a mitochondria‐targeted AMB variant, given reports that AMB disrupts mitochondrial function in tumor cells. The employed gene‐based approach is conceptually aligned with clinically validated cancer gene therapy modalities, as illustrated by the approval of agents such as Gendicine and Imlygic [20, 21], which underscores the translational relevance of evaluating AMB as a suicide gene candidate.

During the analysis of transfected cultures, AMB protein was consistently detected in HEK‐293 T cells but remained undetectable in SK‐MEL‐28 melanoma extracts. This inability to detect AMB in melanoma cells led to two hypotheses: When produced intracellularly, AMB might either selectively kill melanoma cells or undergo preferential degradation within them.

To discriminate between these possibilities, AMB transcript levels and cell viability/apoptosis were evaluated. RNA analysis confirmed that all AMB plasmids were transcribed to comparable levels in both SK‐MEL‐28 and HEK‐293 T cell lines. Functional assays (MTT, caspase 3/7, and transfection quantitation by flow cytometry at different time points) indicated that AMB expression did not induce cell death under the tested conditions, consistent with the absence of detectable AMB protein in tumor cells. These assays were designed to exclude the possibility that AMB‐expressing melanoma cells were being selectively lost from the cultures, which would similarly result in the lack of detectable AMB in SK‐MEL‐28 lysates. The functional data do not support this alternative explanation.

With selective cytotoxicity excluded, post‐translational degradation emerged as the most plausible hypothesis for AMB loss in melanoma cells. Bioinformatic predictions suggested that several lysine residues in AMB could be targets for ubiquitination or SUMOylation. However, site‐directed mutagenesis of these residues failed to rescue AMB protein expression, indicating that blocking these predicted sites is not sufficient to stabilize AMB and suggesting that additional determinants beyond these classical motifs contribute to its turnover.

Partial proteasome inhibition with MG132 enabled AMB detection by western blot in SK‐MEL‐28 cells, supporting the conclusion that the proteasomal system in melanoma cells contributes to AMB degradation under these conditions. This finding is critical, as it highlights a key barrier to using AMB in gene therapy approaches: its intracellular instability when produced directly inside tumor cells. Following proteasome inhibition, the expected molecular weight bands were detected by western blot, confirming that AMB is properly translated but degraded under normal tumor cell conditions. Interestingly, the mitochondria‐targeted AMB displayed both precursor and mature forms, suggesting partial mitochondrial import.

Although previous studies describe AMB as a proteasome inhibitor [12], this work shows that, when expressed intracellularly, AMB itself becomes a proteasome target. This paradox may be related to the absence of its stabilizing disulfide bonds during intracellular expression, which are essential for the correct folding of Kunitz‐type proteins [22, 23]. In addition, the entire C‐terminal region, comprising more than half of the AMB sequence, is intrinsically unstructured, and such unstructured or partially folded regions may expose hydrophobic patches, potentially increasing susceptibility to degradation by the 20S core [24, 25]. This structural characteristic, however, cannot fully account for the differential detectability of AMB in HEK‐293 T and SK‐MEL‐28 cells, since AMB accumulates in HEK‐293 T despite containing the same disordered region. The distinct behaviors of exogenous versus intracellularly synthesized AMB likely reflect the combined influence of these structural features with lineage‐specific differences in cellular proteostasis, which remain to be clarified.

Consistent with this interpretation, differences between tumor and nontumor proteostasis further shape AMB behavior. In HEK‐293 T cells, AMB was detectable and did not induce cytotoxicity. In contrast, melanoma cells degraded the protein. This pattern aligns with reports showing that cancer cells exhibit altered proteostasis and dysregulated proteasome systems compared with nontumor cells [26, 27], including melanoma‐associated changes in proteasome degradation pathways [28, 29, 30]. This altered proteostasis may contribute both to the antitumor selectivity of AMB, as described for the exogenously applied molecule, and to its intracellular instability when produced via gene therapy.

The data suggest that proteasome‐dependent degradation of intracellularly expressed AMB in melanoma cells may result from a combination of tumor‐specific proteostasis features and intrinsic sequence elements that favor recognition and clearance, such as the unstructured C‐terminal region. These mechanisms may act independently or synergistically, leading to the marked difference in AMB stability observed between melanoma and nontumor cells.

## Conclusions

This study evaluated the feasibility of expressing AMB via a suicide gene therapy approach in melanoma cells and assessed its intracellular fate following gene delivery. While AMB displays potent and selective antitumor activity when administered exogenously, the data show that intracellularly expressed AMB does not accumulate in SK‐MEL‐28 melanoma cells due to proteasome‐dependent degradation, whereas it remains detectable in nontumor HEK‐293 T cells. These findings indicate that tumor‐specific proteostasis can act as a barrier to AMB accumulation in a gene therapy context.

This is the first study to investigate AMB as a suicide gene within a gene therapy framework and to identify proteasome‐dependent degradation as a major determinant of its intracellular instability in melanoma cells. By linking this behavior to the structural complexity of AMB, including its Kunitz domain and intrinsically disordered C‐terminal region, the work highlights how such features may render complex protease inhibitors particularly susceptible to clearance in tumor cells. Building on this concept, further studies dissecting the kinetics and pathways of AMB degradation, mapping structural determinants of stability, and testing alternative expression designs, such as rational truncations, will be important to refine the mechanistic understanding and to optimize AMB‐based gene constructs.

By revealing a tumor‐specific bottleneck that limits the intracellular persistence of AMB, this study contributes to the understanding of how structurally complex proteins containing Kunitz domains and unstructured regions behave in the context of gene therapy and points to avenues for improving their performance in cancer models. Addressing the barriers identified here may help determine whether the potent activity of exogenous AMB can be harnessed through gene‐based strategies, potentially adding to the range of molecular tools available for anticancer research.

## Conflict of interest

The authors declare no conflict of interest.

## Author contributions

VDPC conceived, designed and performed experiments, analyzed data, and wrote the manuscript. CDP performed experiments and contributed to experimental design. ARML contributed to data discussion and manuscript revision. MRF conceived the study, performed experiments, and contributed to experimental design. AMCT conceived and supervised the study and contributed to experimental design. All authors revised the manuscript.

## Supporting information


**Fig. S1.**Transfection efficiency of HEK‐293 T cells.

## Data Availability

The data supporting the findings of this study are available from the corresponding author upon request.
