# Supplementary material for: Proteasomal degradation of intracellularly expressed Amblyomin‐X limits suicide gene therapy potential in melanoma cells
Source: FEBS Open Bio. 2026 Apr 2;16(8):1602–14. doi: 10.1002/2211-5463.70224 (PMC13399147; doi:10.1002/2211-5463.70224)
Supplement: Supplementary file 1 — Fig. S1.Transfection efficiency of HEK‐293 T cells. [file FEB4-16-1602-s001.pdf]

Supplementary Figure 1

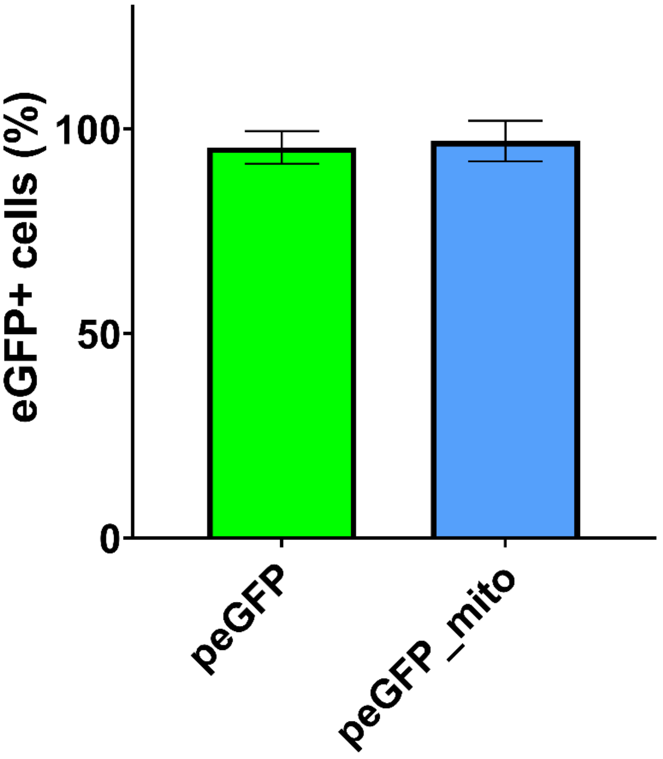

Supplementary Figure 1. Transfection efficiency of HEK-293T cells. Percentage of eGFP-positive HEK-293T cells evaluated by HCS 3 days after transfection. Data are mean ± SD of *n* = 3 independent experiments.
